# Targets for the Induction of Protective Immunity Against Influenza A Viruses

**DOI:** 10.3390/v2010166

**Published:** 2010-01-14

**Authors:** Rogier Bodewes, Albert D.M.E Osterhaus, Guus F. Rimmelzwaan

**Affiliations:** Department of Virology, ErasmusMC, Dr. Molewaterplein 50, 3015 GE, Rotterdam, The Netherlands; E-Mails: r.bodewes@erasmusmc.nl (R.B.); a.osterhaus@erasmusmc.nl (A.D.M.E.O.)

**Keywords:** influenza, protective immunity, pandemic

## Abstract

The current pandemic caused by the new influenza A(H1N1) virus of swine origin and the current pandemic threat caused by the highly pathogenic avian influenza A viruses of the H5N1 subtype have renewed the interest in the development of vaccines that can induce broad protective immunity. Preferably, vaccines not only provide protection against the homologous strains, but also against heterologous strains, even of another subtype. Here we describe viral targets and the arms of the immune response involved in protection against influenza virus infections such as antibodies directed against the hemagglutinin, neuraminidase and the M2 protein and cellular immune responses directed against the internal viral proteins.

## 1. Introduction

Influenza epidemics are the cause of three to five million cases of severe illness every year and approximately 250,000 to 500,000 of these cases are fatal. Epidemics occur during autumn and winter in regions with a temperate climate, while in some tropical countries influenza viruses circulate throughout the year with one or two peaks during the rainy seasons [1]. Mainly the young, elderly and subjects with chronical medical conditions are at risk for developing severe disease after seasonal influenza virus infection. Therefore, the World Health Organization (WHO) recommends annual vaccination of these subjects, which is an effective measure to protect them against influenza and its complications [1].

The genome of influenza viruses consists of eight gene segments of negative sense RNA and since these viruses lack proofreading activity during their replication, they can accumulate mutations under selective pressure. This way, influenza viruses can escape from recognition by virus-neutralizing antibodies that are induced by previous infections or vaccinations. Indeed, the highest degree of variations is observed in the antigenic sites of the hemagglutinin against which virus neutralizing antibodies are directed [2,3]. As a result of this antigenic variation, the influenza vaccine that contains components of three currently circulating influenza viruses (A/H1N1, A/H3N2 and B viruses) has to be updated almost every year to match the circulating strains. Since the selection of the vaccine strains and vaccine production has to be carried out before the start of the influenza season, there is some uncertainty in this prediction and mismatches do occur occasionally. In addition to the small gradual antigenic changes of currently circulating influenza virus strains (antigenic drift), occasionally new influenza viruses of novel subtypes are introduced into the human population. The subtypes of these viruses are defined by the envelope glycoproteins of these viruses, the hemagglutinin (HA) and the neuraminidase (NA). Wild aquatic birds are the natural reservoir of all subtypes of influenza from which there is spillover to other (domestic) birds and mammalian species, like pigs, horses and men [4]. Because antibodies against these viruses are virtually absent in the human population, these viruses may cause pandemic outbreaks of influenza affecting a substantial proportion of the human population. In the last century, three pandemics occurred, which were caused by influenza A viruses of the H1N1, H2N2 and H3N2 subtypes.

Recently, influenza A viruses of swine origin have caused the first pandemic of the 21^st^ century [5]. These new pandemic viruses are the result of the exchange of gene segments originating from human, classical swine and avian-like influenza viruses and have emerged and spread worldwide within a few months [6,7]. As of 30 December 2009 at least 12220 people have been killed due to infection with the influenza A/H1N1(2009) virus [8]. Since not all fatal cases are reported, the real number of fatal cases is most likely much higher.

In contrast to the efficient human-to-human transmission and the rapid spread of the new influenza A/H1N1 viruses, the highly pathogenic avian influenza A viruses of the H5N1 subtype, first detected in humans in 1997 [9,10] are transmitted from human-to-human inefficiently so far, although clusters of human-to-human transmission have been reported [11,12]. However, of the 438 human cases that have been reported since 2003, 60% had a fatal outcome [13] and therefore it is feared that these viruses may adapt and become pandemic in the future. For example, mutations in the receptor binding site may allow these viruses to use the receptor present on most cells of the human tracheal and bronchial epithelium (sialic acid-α-2,6-Gal-terminated saccharides; α-2,6-SA) in addition to the avian receptor (α-2,3-SA), which is a prerequisite for replication in the upper respiratory tract and efficient transmission between humans [14].

In addition to avian A/H5N1 viruses and the new A/H1N1 viruses of swine origin, also influenza A viruses of other subtypes have crossed the species barrier and have infected humans recently. In 2000, an avian influenza A/H9N2 virus infected two children in Hong Kong causing only mild disease [15] whereas in 2003, during an outbreak of highly pathogenic avian influenza A/H7N7 in poultry in The Netherlands 89 humans were infected of which one died [16].

Because of the continuous threat of influenza pandemics and epidemics, there is considerable interest in the development of vaccines that can induce protective immunity against these viruses. Since influenza viruses are “moving targets”, ideally new vaccines should induce broad protective immune responses against multiple subtypes of influenza A viruses. In the present review, we discuss influenza virus proteins as targets for the induction of protective immunity against these viruses with emphasis on those proteins that are targets for the induction of heterosubtypic immunity.

## 2. Influenza viruses and their proteins

Influenza A viruses are enveloped single stranded negative sense RNA viruses with a genome consisting of eight gene segments encoding eleven different proteins. These eight RNA segments are independently encapsidated by the nucleoprotein (NP) and associated with the polymerase proteins PB1, PB2 and PA, which together form the ribonucleoprotein complex [17]. The polymerase proteins are responsible for replication and transcription of vRNA and mRNA respectively [18]. The matrix protein M1 functions as a spacer between the RNP complexes and the viral envelope and interacts with both. The viral envelope is derived from the host cell membrane. Two major surface glycoproteins (HA and NA) are inserted and protrude from the viral envelope. The HA is the receptor binding protein, facilitating attachment of the virus particle to the host cell. The HA is synthesized as a precursor polypeptide HA_0_ which requires proteolytic cleavage into HA_1_ and HA_2_ subunits before it becomes functional and virus particles can infect cells. The HA_1_ subunit contains the receptor-binding pocket and the relatively conserved HA_2_ unit constitutes the stem region containing the fusion peptide. This fusion peptide plays an important role in pH-dependent fusion of the viral envelope with the endosomal vesicle.

By acting as a receptor-destroying enzyme, the NA plays an important role in the virus replication cycle after budding of new viruses from the infected cell. NA cleaves sialic acid residues, which promotes release of newly produced virus particles from the infected cell.

The minor envelope protein, M2, is the result of alternative splicing of mRNA encoding M1. It functions as an ion channel and facilitates the influx of H+ ions into the virus particle, resulting in uncoating of the RNP complex and their release into the cytoplasm of the cell, which is a crucial step in the replication cycle. M2 is the target for the antiviral drug amantadine.

Two non-structural (NS) proteins are also expressed in the infected cell, NS1 and NS2. NS1 is a multifunctional protein and is known for antagonizing the host cell IFN production and its activity [18]. NS2 is involved in nuclear transport of RNP complexes. Recently, the eleventh viral protein was identified which is transcribed from an alternative reading frame of PB1 (PB1-F2) [19]. Most likely, this protein plays a role in promoting apoptosis of the infected cell.

As for other virus infections, influenza viral proteins are degraded in the cytosol of the infected cell by the proteasome into peptides. These peptides are transported to the endoplasmatic reticulum where they can bind to MHC class I molecules. The MHC class I peptide complexes are subsequently transported to the surface of the infected cells where they can be recognized by virus specific CD8+ T cells (see below).

## 3. Immunity to influenza viruses

Infection with influenza virus does not induce lifelong protective immunity against influenza infection in humans, even not against infection with the same subtype. The main reason for this is that influenza A viruses continue to circulate as antigenic drift variants, that have accumulated mutations in antigenic sites of the HA molecule that are recognized by virus neutralizing antibodies. However, the induction of antibodies of the proper specificity will afford strain-specific protection and this strain-specific immunity can be very long lasting. Recently, it was demonstrated that humoral immunity against the 1918 influenza A/H1N1 virus was still present in some individuals that were born before 1918, nearly 90 years after the start of the pandemic [20]. In addition, a substantial proportion of the elderly have potentially protective antibody titers against the influenza A/H1N1(2009) virus, most likely as a result of historical exposure to a similar virus [21].

Since subtypes of the influenza viruses are defined by the absence of mutual cross-reactivity of subtype specific antibodies [22], antibodies to one subtype will not afford protection against infection with an influenza virus of another subtype. However, it has been demonstrated that infection with an influenza A virus can induce a certain degree of protective immunity against infection with an influenza A virus of another subtype, although infection cannot be prevented [23]. This so-called heterosubtypic immunity was first described more than four decades ago [24]. For example, infection of mice with influenza A/H3N2 or A/H9N2 viruses ameliorated the clinical course of infection with highly pathogenic influenza A/H5N1 viruses considerably and reduced mortality rates [25,26]. Heterosubtypic immunity induced by infection has shown to be long-lasting (18 months) in the ferret model, which is the gold standard model for human influenza A virus infections [27]. The immunologic basis underlying heterosubtypic immunity has been the topic of numerous studies [23]. Experiments in multiple knock-out and transgenic mouse models have shown that virus-specific CD4+ T cells (T helper cells), CD8+ Cytotoxic T cells (CTL), mucosal antibodies (IgA) and B cells can contribute to heterosubtypic immunity [28–33]. Especially cell-mediated immune responses directed to conserved proteins of influenza A viruses are believed to play an important role.

There is also evidence that infection with an influenza A virus can induce heterosubtypic immunity in humans [34–36]. For example, individuals that had experienced an infection with an influenza A/H1N1 virus before 1957 less likely developed flu during the H2N2 pandemic of 1957 [35].

## 4. Influenza A virus vaccines

Most influenza virus vaccines that are currently used against seasonal influenza viruses and against the influenza A/H1N1(2009) virus are prepared by infecting embryonated chicken eggs with influenza virus vaccine strains. Subsequently allantoic fluids of infected eggs are harvested and the egg-derived virus is purified. Depending on the vaccine manufacturer, influenza viruses are inactivated with formaldehyde or ß-propiolactone to prepare a whole inactivated influenza virus vaccine or treated with a detergent to prepare a split or subunit influenza vaccine. Subunit vaccines are, after treatment with a detergent, further purified to remove all viral proteins and lipids except the HA and NA. To obtain high yields of influenza virus antigens after infecting chicken eggs, reassortant viruses are prepared by infecting embryonated chicken eggs simultaneously with a selected epidemic strain and an egg-adapted laboratory strain, typically influenza A virus A/PR/8/34. Reassortant viruses that carry the HA and NA of the epidemic strain and that grow to high virus titers are selected and used as vaccine strain.

Influenza virus vaccines can also be prepared by growing viruses in cell cultures or by using live attenuated influenza viruses. Cell culture-derived influenza virus vaccines are prepared essentially with the same procedure that is used to prepare egg-derived influenza virus vaccines, except that cells are used to propagate viruses. Live attenuated viruses are typically attenuated by adapting viruses to replicate at lower temperatures. Cold-adapted vaccine strains are subsequently prepared by reassortment with selected epidemic strains to ensure that the vaccine strains contain the proper HA and NA.

Currently, alternative formulations and methods to prepare influenza virus vaccines are in various stages of development or already licensed. Examples are the production of vaccine strains by reverse genetics, the use of virosomes and virus-like particles and the expression of viral genes in recombinant baculoviruses or modified vaccinia viruses [37–41].

## 5. Viral targets for the induction of humoral immunity

### 5.1. Hemagglutinin

It has been well documented that the induction of HA specific antibodies correlate with protection against infection, provided that they have the proper strain-specificity [42]. These antibodies can neutralize the virus by binding to the region responsible for binding of the HA molecule to its receptor on host cells. This way, binding of virus to the host cell is prevented efficiently. Therefore the induction of HA antibodies that block receptor binding is used as a correlate of vaccine efficacy and vaccines are registered every year when they fulfill the minimal EMEA/FDA requirements according to the serological outcome of vaccination and potency of the vaccine (>15μg HA per vaccine strain). To this end, pre- and post vaccination sera are tested in the hemagglutination inhibition (HI) assay to assess the HI antibody seroconversion rates and the proportion of study subjects that obtained protective antibody levels. Antibody titers ≥40 in this HI assay are considered protective [42].

As outlined above, HA-specific antibodies to one subtype per definition will not cross-react with another. Sixteen subtypes of HA have been identified so far [43]. These subtypes are discriminated by double immunodiffusion assays using hyperimmune animal sera, confirming the mutual lack of cross-reactivity between subtypes [22]. Furthermore, the structure of antigenic sites vary among different subtypes, as it was demonstrated that the structures of antigenic sites of H5 [44,45], H9 [46] were different from H1, H2 and H3 subtypes. Five antigenic sites of the H3 subtype have been identified, mainly in the globular head region [47–50]. Antigenic sites of H1 and H2 have also been characterized by the identification of amino acid substitutions in the HA sequence [51,52].

Although antisera against one subtype do not cross-react with an other subtype, monoclonal antibodies have been described that do cross-react with various HA subtypes [53,54]. Passive immunization with these HA-specific antibodies afforded protection against viruses of various subtypes [55–62]. More recently, a monoclonal antibody was generated that recognizes a common epitope on the globular head region of HA. This antibody inhibited virus binding to host-cell receptors and when administered to mice, protected against challenge infection with influenza viruses of the H1 and H3 subtypes [63].

Using combinational libraries built from B cells obtained from subjects recently vaccinated against seasonal influenza, a number of human antibodies were derived that displayed an unexpected broad reactivity with various subtypes of influenza virus, which were neutralized by these antibodies. The binding region of these monoclonal antibodies is located in the conserved region of the HA stem domain. Their prophylactic and therapeutic efficacy against H5N1 and H1N1 influenza A viruses was demonstrated in mice [64]. Thus, although the serum antibody response to influenza virus HA molecules is subtype specific and even specific for individual variants within a subtype without cross-reactivity with other strains, some conserved epitopes do exist and antibodies against these epitopes may exert biological activity. It is unclear whether the antibody response to these conserved epitopes contributes to heterosubtypic immunity. It was demonstrated that mice intranasally vaccinated with an influenza A/H3N2 vaccine adjuvanted with an E. coli heat labile enterotoxin were protected against infection with an influenza A/H5N1 virus [65]. Using B-cell deficient IgH-6-/- and B2m mice, it was demonstrated that this type of protection was dependent on the presence of B-cells, most likely at the site of infection.

### 5.2. Neuraminidase

The NA plays an important role in the virus replication cycle after budding of new viruses from the infected cell, by cleaving sialic acid molecules from cellular receptors on infected cells and newly produced virus particles, thus acting as a receptor-destroying enzyme. It has been demonstrated that antibodies against NA can inhibit its enzymatic activity [66–68]. Since this biological activity only can take place in a late step of the virus replication cycle, NA specific antibodies cannot prevent infection, like HA-specific antibodies [69–71]. However, the induction of NA-specific antibodies can markedly reduce virus replication by inhibiting the release of newly produced virus particles and shorten the severity and duration of illness [69,72–74]. Furthermore, it has been demonstrated that NA can play a role during the entry stage of influenza virus infection of human airway epithelial cells *in vitro.* This suggests that antibodies that bind to NA have an impact during an early stage of the infection cycle. However, the exact mode of action of this function of NA and the relative contribution of antibodies that blocks this function to protective immunity is unknown [75].

NA-specific antibody responses have been detected in humans after vaccination with inactivated vaccines [66,76], which may also contribute to the clinical vaccine effectiveness of influenza vaccines. Although NA and HA are both immunogenic, intact influenza viruses induce a stronger antibody response to the HA than to NA as result of antigenic competition [67,68,77–79].

Recently, it was demonstrated that vaccination of mice with a DNA vaccine from which the NA gene of a contemporary human H1N1 strain was expressed conferred protection against infection with influenza viruses of both the H1N1 and H5N1 subtype [80]. Furthermore, it was shown that in human sera, antibodies against the NA of human influenza viruses were present that also inhibited the enzymatic activity of NA of the N1 subtype derived from avian influenza A viruses [80]. This data indicated that induction of NA in vaccines may broaden their protective potential against viruses with unrelated HA subtypes.

### 5.3. M2 protein

The M2 protein is a membrane protein with ion channel activity and plays an important role in the virus replication cycle. Compared to HA and NA, it is a minor antigen on mature virions, however its expression in virus-infected cells can be readily detected [81–83]. The first evidence that antibodies to the M2 protein have antiviral activity was demonstrated *in vitro* using a mouse monoclonal antibody directed to M2 [84,85]. Administration of M2-specific monoclonal antibodies intravenously to recipient mice inhibited influenza A virus replication in infected animals. Therefore, M2 was considered a promising target for the induction of protective immunity against influenza A viruses. Since this protein is very conserved, even between different subtypes of influenza A virus originating from various animal species, it was also considered as a vaccine candidate that could induce broadly protective antibody responses [86,87]. Indeed, hyperimmunization with vaccines based on the M2 protein or its 23 amino acid ectodomain (M2e) induced antibodies that protected experimental animals against infection with viruses of various subtypes (for review see [88]). To increase the immunogenicity of M2e it was coupled to carriers such as the Hepatitis B virus like particles [87–90]. The mode of action of vaccine-induced M2e is probably not direct neutralization of virus, but involves antibody dependent cellular cytotoxicity by NK cells which contribute to the elimination of virus-infected cells [32,86,89,91].

There is concern that the induction of M2 specific antibodies in the population after large-scale use of an M2 based vaccine might increase the selective pressure on this protein, which could drive escape from recognition by these antibodies. Although escape mutants were observed after infection of SCID mice treated passively with M2 antibodies, the likelihood of the emergence of escape mutants in vaccinated mice is low [92].

In post-infection sera of humans, antibodies to M2 are virtually absent [93] which indicates that these antibodies most likely do not contribute to infection-induced heterosubtypic immunity. Adoptive transfer experiments with serum from infected mice confirmed that M2 antibodies did also not contribute to heterosubtypic immunity [29].

### 5.4. Nucleoprotein

Upon infection with influenza A virus, also antibodies are induced against other structural proteins including the nucleoprotein (NP) [94]. Antibodies against the NP can also be induced by vaccination. However, these antibodies are considered non-protective since passive transfer of serum of mice vaccinated with recombinant NP vaccines to SCID mice did not protect these mice [95]. In contrast, recent studies have shown that rNP immunization reduced morbidity and virus replication after influenza virus infection. Furthermore, NP-immune serum transfer to naïve recipient mice conferred this protection in an antibody dependent manner [30].

It was also demonstrated that the induction of non-neutralizing antibodies including those with specificity for NP contributed to heterosubtypic immunity. Although NP specific antibodies cannot neutralize influenza virus, indirectly they may contribute to protective immunity by promoting virus-specific CD8+ T cell responses and the production of VN- antibodies [96]. The formation of immune complexes with anti-NP antibodies leading to DC maturation and Th1 cytokine production may also contribute to heterosubtypic immunity against influenza [97].

## 6. Viral targets for the induction of cellular immunity

### 6.1. Hemagglutinin and neuraminidase

The CD4+ T helper cell response after influenza virus infection and vaccination is multi-specific and also HA- and NA-specific CD4+ T cells are induced (for review see [98]). CD4+ T cells are crucial for the optimal activation and early expansion of B cells, for the initiation and maintenance of germinal center reaction and the generation of long-lived plasma and memory B-cells [99–102]. They also play a role in the control of virus infection by promoting CD8+ cytotoxic T cell responses [103–106].

Furthermore, it has been suggested that CD4+ T cells also can attack virus-infected host cells directly [107]. It is of interest to note that amino acid substitutions have been identified in the HA molecule during influenza virus evolution that do not affect recognition by virus-specific antibodies, but that are associated with escape from recognition by virus-specific CD4+ T cells [108]. These findings suggest that certain T helper cell epitopes of influenza virus are under selective pressure similar to those recognized by antibodies [3] or virus-specific CD8+ CTL [109]. On the other hand, it has been demonstrated that also more conserved epitopes are located on the HA leading to cross-reactive T cell responses [110–112].

In contrast to CD4+ T cell responses, hardly any virus-specific CD8+ T cells were directed against the HA and NA in naturally infected study subjects [111] (see also Table 1). Most likely this is the result of intracellular trafficking of HA into the ER, entering the endogenous pathway of antigen processing inefficiently. This takes place in the cytosol where other viral proteins are present more abundantly. Nevertheless, some epitopes have been identified in HA molecules recognized by virus-specific human and mouse CD8+ CTL [113–117].

### 6.2. Structural proteins, polymerases and NS proteins

The virus-specific CD4+ T cell responses after influenza virus infection is directed against a variety of proteins, including NP, M1, PB1, PB2, PA and NS1. Also the CD8+ T cell responses are largely directed against these proteins and a large number of epitopes have been identified which are recognized by virus-specific CD8+ T CTL (Table 1). As shown in Table 1, the number of both CD4+ and CD8+ T cell epitopes present on the respective influenza A virus proteins vary. Numbers were obtained from the Immune Epitope Database [118,119].

The main function of CD8+ T cells is the elimination of virus-infected cells through the release of perforin and granzyme or Fas/FasL interaction. In addition, activated CD8+ T cells produce cytokines like IFN-γ and TNF-α which can modulate the immune response.

The majority of CTL epitopes is fully conserved between intra-subtypic variants of influenza A viruses. However, it has been demonstrated that some immunodominant CTL epitopes display variability that is associated with escape from recognition by specific CTL [109,120–122]. In most cases, mutations are fixed during viral evolution, which indicates that these CTL exert selective pressure. Indeed, a lower ratio between synonymous and non-synonymous (Ds/Dn) mutations was observed in CTL epitopes located in the NP than in the rest of the protein [120]. It was demonstrated that naturally occurring mutations in CTL epitopes affected the human *in vitro* CTL response significantly [123,124]. In contrast, other epitopes are fully conserved, including the HLA-A*0201 restricted immunodominant epitope from the matrix protein M1_58-66_. Since HLA-A*0201 has a high prevalence in the human population, the selective pressure on this epitope must be high. The lack of variation in this epitope was explained by functional constraints, since mutations were not tolerated without the loss of viral fitness [125,126].

Many influenza A virus epitopes are also conserved between subtypes, which suggested that virus-specific CTL play a role in heterosubtypic immunity [127–130]. Indeed, adoptive transfer and depletion experiments in mice have demonstrated that CD8+ CTL contribute to protective immunity against heterosubtypic strains of influenza A virus [131–136]. Especially when serum antibodies of the proper specificity are not present, virus specific CTL may be an important correlate of protection against the development of severe disease. Also in humans it was demonstrated that the presence of cross-reactive CTL inversely correlated with the extent of viral shedding in the absence of antibodies specific for the strain used for experimental infection of the study subjects [34]. Thus, cross-reactive T cell responses directed against conserved epitopes after infection may afford some degree of protection against influenza viruses of other subtypes. Therefore, the use of conserved proteins like NP and M1 for the induction of cross-protective CTL responses may be a promising approach for the development of “universal” influenza vaccines. For example, the use of live attenuated vaccines, the use of adjuvants like ISCOMS that stimulate CD8+ T cell immunity or novel generations of influenza vaccines, like viral vector vaccines, may be attractive alternatives [137–141]. Examples of vector vaccine production platforms are the recombinant replication deficient adenoviruses, poxviruses and Newcastle disease virus vectors, which have been shown to induce protective immunity to influenza viruses efficiently [142–146]. These vaccine candidates facilitate antigen processing and presentation by the endogenous route, which is a prerequisite for the efficient induction of CTL responses [147].

The induction of cross-reactive CTL responses by natural infections also may have epidemiological implications. It was demonstrated that subjects who experienced a prior A/H1N1 infection, less likely developed influenza during the A/H2N2 pandemic of 1957 [35]. Furthermore, it is of interest to note that especially younger individuals are at risk for severe disease upon total outcomes of H5N1 and new H1N1 influenza virus infections [148,149]. Although confounding factors cannot be excluded, younger individuals may have been less exposed to seasonal influenza A viruses and consequently have developed less CTL immunity than older subjects.

## 7. Concluding remarks

**Figure 1.**
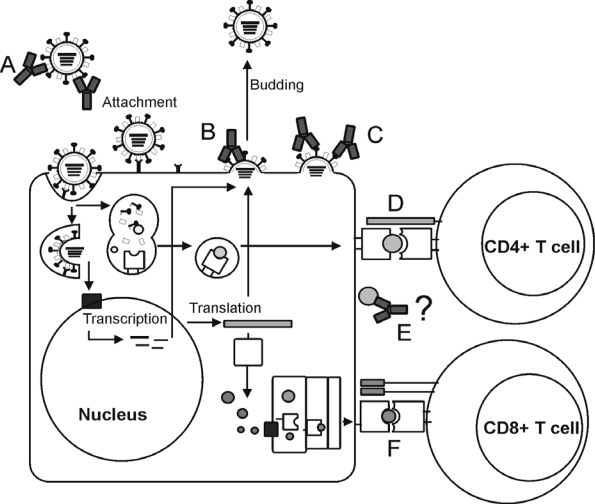
Overview of the targets of the immune system for the induction of protective immunity against influenza. **(A)** HA-specific antibodies can bind to the HA on viruses and prevent infection of cells. **(B)** M2e specific antibodies can bind to M2e on virus-infected cells and induce antibody-dependent cell-mediated cytotoxicity (ADCC). **(C)** NA specific antibodies inhibit enzymatic activity of NA and thus further spread of newly produced virus particles. **(D)** Pathogens and proteins are broken down into peptides within acidified endosomes and these peptides bind to MHC Class II, MHC Class II peptide complexes are subsequently transported to the surface of the cell for recognition by CD4+ T cells. **(E)** The mode of action of NP-specific antibodies is largely unknown. **(F)** Influenza viral proteins are degraded in the cytosol of the infected cell by the proteasome into peptides that are transported to the endoplasmatic reticulum where they can bind to MHC class I molecules. The MHC class I peptide complexes are transported to the surface of the infected cells for recognition by CD8+ T cells, which subsequently eliminate the infected cell.

After infection with influenza viruses various arms of the adaptive immune system are activated (Figure 1). In Table 2, the viral targets for the induction of protective antibody responses and their mode of action are shown.

The envelope proteins are the most important targets for the induction of virus-specific antibodies. The induction of sufficiently high titers of HA-specific antibodies affords sterilizing immunity against infection provided that these antibodies have the proper specificity for the strains causing the infection. The induction of NA specific antibodies also contributes to protective immunity, but since these antibodies interfere with a late step in the virus replication cycle they cannot prevent infection. Furthermore, NA-specific antibodies need to have specificity for the strain causing the infection, like HA-specific antibodies. In contrast, M2-specific antibodies induced after hyperimmunization or passively administered, afford protection against multiple influenza virus strains and even against multiple subtypes of influenza A virus, since this protein is highly conserved. Also M2-specific antibodies do not afford sterilizing immunity since their most important mode of action is through ADCC after binding to infected cells expressing M2 on their surface. The mode of action and the effectiveness of non-neutralizing NP-specific antibodies are not fully understood although it has been demonstrated after hyperimmunization that they afford some protection.

In addition to the systemic and/or mucosal antibody responses, also virus-specific T cells contribute to protective immunity against infection (Table 3). T helper cells, which are directed to virtually all viral structural proteins and polymerases, provide the essential signals for the activation and differentiation of both B-cell responses and CD8+ T cell responses. In addition, it has been suggested that they are able to attack virus-infected cells directly. The latter function is especially executed by virus-specific CTL, which preferentially recognize internal structural proteins like NP and M1. Since these proteins are highly conserved between subtypes, CTL responses are cross-reactive and contribute to heterosubtypic immunity.

In the design and development of vaccines against newly emerging variants of influenza viruses or novel pandemic strains, the induction of antibodies directed to the HA (and to a lesser extent, NA) of these viruses is preferable. However, the development of such vaccines is time-consuming and therefore they cannot always be delivered in a timely fashion. The induction of immunity to conserved viral antigens, like antibodies to M2 or cell-mediated immunity to NP or M1, may be an attractive approach for the development of more universal vaccines. These could be used as standalone vaccine or they may broaden the protective potential of existing vaccines. However, in clinical trials with these vaccine candidates, it will be difficult to demonstrate the improved protectiveness of vaccines against heterosubtypic strains. Most likely one has to rely on surrogate markers of this type of immunity like demonstrating the presence of M2e-specific antibodies and CTL that cross-react with heterosubtypic strains of interest *in vitro*. More research is required to establish that the induction of immunity against these conserved antigens is also a correlate of protection in humans. Results obtained so far in animal models are promising.

## Figures and Tables

**Table 1.** Human CD8+ and CD4+ T cell epitopes on the influenza A virus.

**Protein**	**Number of MHC Class I epitopes**	**Number of MHC Class II epitopes**
HA	6	46
NA	5	13
M1	15	31
M2	2	0
NP	25	32
PA	8	2
PB1	21	5
PB2	1	1
NS1	2	1
NS2	2	0

The number of CD4+ and CD8+ T cell epitopes were obtained from the Immune Epitope Database [119] using the following settings: Source organism: Influenza A, Host organism: Homo sapiens, Immune recognition context: T cell response and MHC class I/II binding. Multiple epitopes on the same location counted as one, all HLA types included.

**Table 2.** Viral targets for the induction of protective antibodies.

**Viral antigen**	**Mode of action**	**Comments**
HA	Prevents virus attachment to host cells	- Antibodies must have proper specificity - strain specific
NA	Inhibits enzymatic activity of NA and spread of virus	- Antibodies must have proper specificity
M2	Induction of antibody-dependent cell-mediated cytotoxicity (ADCC) and elimination of infected cells	- M2 is highly conserved - Hyperimmunization induces cross-protective immunity
NP	Largely unknown, complex formation?	- Non-neutralizing - Mode of action and effectiveness unknown

**Table 3.** Viral targets for the induction of protective T cell responses.

**Viral antigens**	**Type of response**	**Comments**
All viral proteins	CD4+ T helper cell response	- Polarization (Th1/Th2) dependent on antigen delivery - Essential for B-cell and CD8+ CTL responses - Direct action against infected cells - HLA restricted
PB1/PB2/PA/NP/M1/M2/NS1	CD8+ CTL response	- Key role in elimination of infected cells - Cytokine production - HLA restriction dictates magnitude of response - Only marginal response to HA
